# GoldenBac: a simple, highly efficient, and widely applicable system for construction of multi-gene expression vectors for use with the baculovirus expression vector system

**DOI:** 10.1186/s12896-020-00616-z

**Published:** 2020-05-12

**Authors:** Jana Neuhold, Katharina Radakovics, Anita Lehner, Florian Weissmann, Maria Queralt Garcia, Mari Carmen Romero, Nicholas S. Berrow, Peggy Stolt-Bergner

**Affiliations:** 1 Protein Technologies Facility, Vienna BioCenter Core Facilities, Dr. Bohr-gasse 3, 1030 Vienna, Austria; 2 Research Institute of Molecular Pathology, Campus Vienna Biocenter 1, 1030 Vienna, Austria; 3 Protein Expression Core Facility, Institute for Research in Biomedicine (IRB) Barcelona, The Barcelona Institute of Science and Technology, C/ Baldiri Reixac 10, 08028 Barcelona, Spain

**Keywords:** Golden Gate cloning, Baculovirus expression, Multi-gene expression, Protein complex, Co-expression

## Abstract

**Background:**

Recombinant protein production and purification of large protein complexes in eukaryotes requires efficient methods to generate multi-gene expression constructs, where each individual gene is under the control of its own promoter and terminator. Current methods are based either on serial rounds of combination of several vectors containing loxP sites via the Cre-lox technology, or on multiple rounds of gene combination via PCR or other methods. These methods are multi-step, have lower efficiencies than single gene cloning, and may require laborious processes to verify that all genes of interest are present in the final product. Here, we describe a rapid and simple Golden Gate-based system for the generation of multi-gene expression constructs compatible with baculovirus expression vector systems (BEVS) using either Tn7 transposition or KO1629-based homologous recombination, which we refer to as “GoldenBac”.

**Results:**

This method is based on the construction of a series of vectors containing a promoter-gene of interest-terminator cassette flanked by cleavage sites of the BsaI type IIS restriction enzyme. This series of vectors can be cut by BsaI to excise cassettes with unique overhangs. In the same reaction, the cassettes are then ligated in the correct sequence in a final destination vector to generate multi-gene expression constructs containing 2–15 genes. Individual expression constructs can therefore be combined into a single vector in a single reaction, with over 90% efficiency when combining up to 14 expression cassettes. We demonstrate successful construction and expression of three different co-expression systems, the proteosomal lid complex, the anaphase promoting complex/cyclosome (APC/C), and a series of constructs used to test the effect of chaperone co-expression on the solubility of the HOIP protein.

**Conclusions:**

This robust, single-step cloning system provides an easy-to-use method for generation of multi-gene expression constructs for both transposition and homologous recombination-based baculovirus systems, making this technology available across all laboratories using baculovirus expression systems. This highly efficient and simple method allows for rapid incorporation of multi-gene expression cloning into the standardized service portfolio of protein production facilities and can also easily be adopted by any laboratory for routine generation of multi-gene baculovirus constructs.

## Background

In order to uncover the molecular mechanisms underlying cellular processes, it is necessary to analyze not just single proteins, but multi-subunit protein complexes. In the past 5 years, we have experienced a paradigm shift in the ability to determine atomic resolution structures of even small protein complexes [1]. As methods to analyze these molecular machines on a structural and functional level advance, efficient methods are required to generate pure and homogeneous recombinant protein complexes. One of the most successful systems for recombinant protein production of eukaryotic proteins is the baculovirus expression vector system (BEVS), in which an expression cassette is incorporated into a baculovirus genome and that virus is then used to infect insect cells, which subsequently produce high levels of the protein of interest. The BEVS has been successfully used to generate recombinant protein complexes of higher quality than those obtained from endogenous sources [2, 3]. While insect cells can be simultaneously infected with several baculoviruses, this approach quickly reaches its limit when more than 3 individual viruses are required for co-expression. As many protein complexes of structural interest can contain more than 10 components, in order to take advantage of the BEVS for the production of high-quality recombinant protein complexes, efficient methods are required for generation of multi-gene expression constructs.

The past 10–15 years have also seen a revolution in the application of molecular biology techniques to multi-gene expression, in particular in the context of synthetic biology, but also with relevance for recombinant protein production. The first widely available system for generation of multi-gene constructs for baculovirus-based recombinant protein production was the MultiBac™ system [4]. MultiBac™ took advantage of the Cre-lox technology to combine individual plasmids containing one or more promoter-gene-terminator cassettes. Additionally, the MultiBac™ system introduced a yellow fluorescent protein (YFP) marker onto the baculoviral bacmid as a useful feature for easy monitoring of successful virus production/infection [5]. More recently, a system called biGBac, which is based on Gibson cloning, has been developed, which allows for parallel generation of constructs containing up to 25 individual genes in two sequential steps [2]. Additional methods, including systems based on USER cloning and BioBricks have also been developed, illustrating the requirement of many laboratories for efficient generation of multi-gene expression constructs for use with the baculovirus system [6, 7].

In our facilities, we have attempted to use existing systems, including both MultiBac™ and biGBac, to generate multi-gene expression constructs for our users. We found the systems to be too inefficient (< 20% of clones screened contain a correctly assembled construct; see Table 2) and too cumbersome due to the requirement for multiple rounds of PCR and sequence validation steps for routine use in the context of a facility. We therefore set out to develop a simple, one-step, robust system of high efficiency that we could use to reliably generate constructs in a time and therefore also cost-efficient manner. In addition, the more recent multi-gene expression systems have been created for generation of the baculoviruses through Tn7 transposition, as in the Bac-to-Bac system, originally sold by Invitrogen [8, 9]. However, many laboratories are now using homologous recombination of an appropriate expression plasmid with linearized bacmid (based on the baculoviral ORF1629 gene knockout (KO1629) [10];) in insect cells to generate recombinant baculoviruses. These linearized bacmids are marketed commercially by various suppliers (*Flash*Bac: Oxford Expression Technologies, BacMagic: Merck Millipore and BaculoGold: BD Biosciences). Although the generation of baculoviruses via the homologous recombination/KO1629-based route is gaining popularity, many existing systems, such as biGBac, offer no route of entry to this method of virus generation.

As the basis for a high-efficiency multi-gene cloning system, we chose Golden Gate cloning [11]. Golden Gate cloning has been used most often in synthetic biology to generate large constructs containing many genes/transcriptional units in a certain metabolic pathway [12]. Golden Gate cloning has also been used in the MoClo and GreenGate systems to generate expression vectors for *Agrobacterium* transformation in *A. thaliana*, and is used in many CRISPR/Cas9 vector systems [13, 14]. This method is based on the properties of type IIS restriction enzymes, which cleave DNA at a defined distance outside their non-palindromic recognition site. This allows for the possibility to generate several DNA fragments containing short, unique single-stranded overhangs even after cleavage with the same enzyme. By combining such an enzyme with a DNA ligase, it is possible to cut many DNA fragments with the same enzyme and ligate them to each other in a defined sequence, all in a single tube reaction.

Our previous experience using the Golden Gate cloning system for generation of genome engineering constructs led us to hypothesize that this cloning methodology had the high efficiencies necessary for a simple and cost-effective method for generation of multi-gene constructs for recombinant protein production. We have created the GoldenBac set of vectors, which consists of two systems of 16 vectors to be used for generation of multi-gene expression constructs for the baculovirus expression vector system. One set of vectors is adapted for Tn7 transposition, whereas the second set is compatible with both Tn7 transposition and homologous recombination. We show that the GoldenBac system can be used to assemble up to 15 gene expression cassettes in a single step with efficiencies of up to 90%. We further show that GoldenBac compares favorably with biGBac, and can be easily applied to any expression project requiring screening of multiple multi-gene expression constructs.

## Results

### GoldenBac allows for highly efficient multi-gene expression in insect cells

**Fig. 1:**
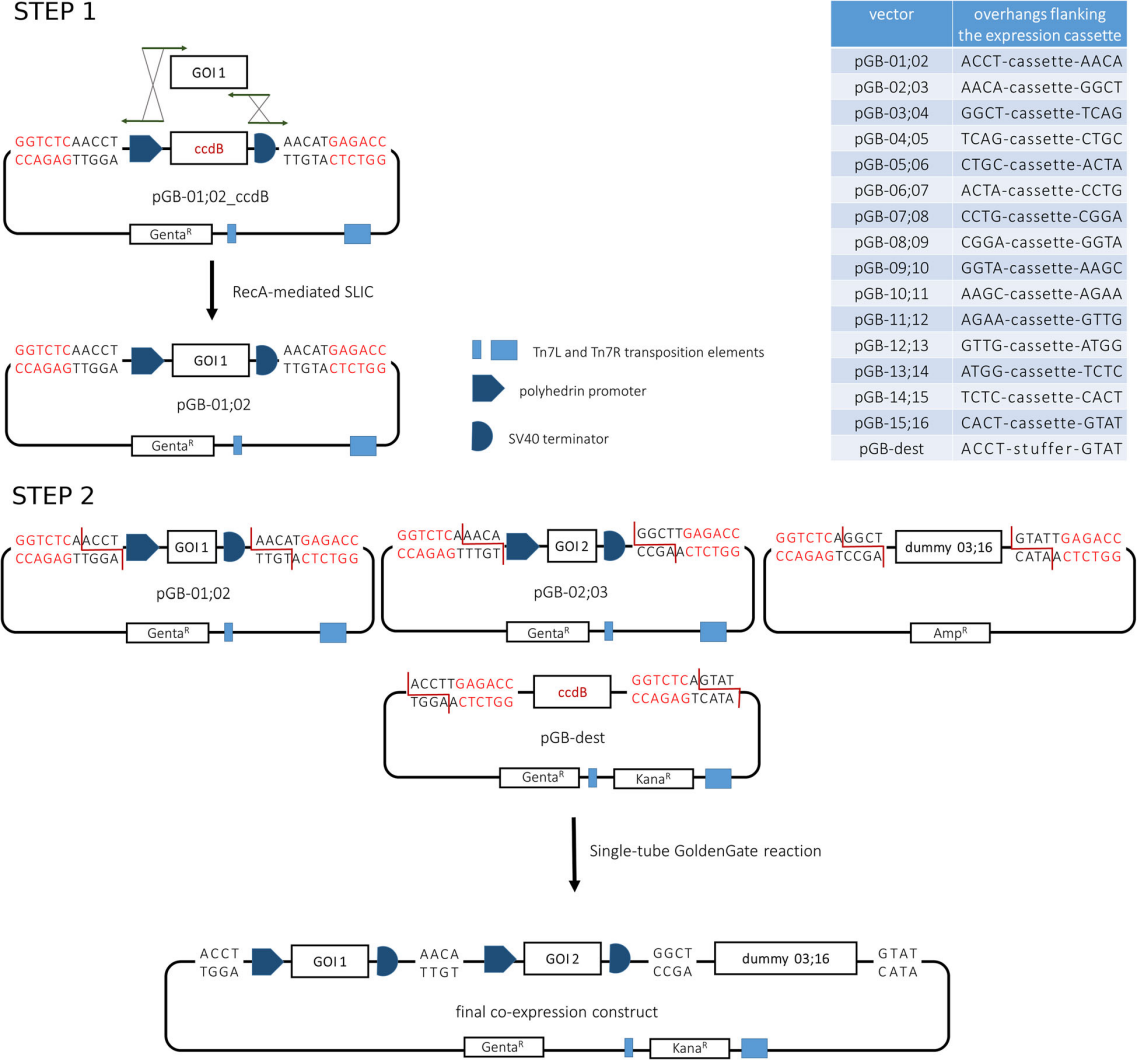
Overview of the GoldenBac strategy. In the first step, single targets are PCR-amplified using primers with extensions homologous to the primers used for PCR-linearization of the selected vector and the purified PCR products are combined into single expression construct via a RecA-mediated Sequence and Ligation Independent Cloning or In-Fusion cloning strategy. Small tags for purification or detection can be easily included on the primer extensions and internal BsaI recognition sites can be removed in this step by “fragmentation” of the gene. In the second step, expression cassettes are released from the single expression constructs upon cleavage with the BsaI restriction enzyme (recognition sites shown in red) and simultaneously ligated into the destination vector, based on the inter-compatible overhangs flanking the cassettes. Single or multiple positions can be encompassed by short sequences flanked by BsaI sites with fitting overhangs, called dummies. Selection against kanamycin, present only on the destination vector, will result in recovery of the final co-expression construct with high efficiency, both due to negative background selection of the empty destination vector with the *ccdB* spacer and due to the enrichment of final product by self-removal of the BsaI site

In order to generate multi-gene expression cassettes, the baculovirus expression vector pACEBac1 was modified by adding two restriction sites for the type IIS enzyme BsaI; one upstream of the promoter, and one downstream of the terminator. Cleavage of the resulting vector with the BsaI enzyme then results in excision of a promoter-gene of interest-terminator cassette containing unique 5′ and 3′ overhangs, as shown in Fig. 1. The BsaI site upstream of the promoter was positioned in front of the available PmeI restriction enzyme site so that it will be transferred with the expression cassette in order to facilitate screening of final constructs via restriction digest. A spacer bearing the sequence of the lethal *ccdB* gene was included between the promoter and terminator to allow for negative background selection during the first cloning step [15].

A series of entry vectors was then created in the same manner, resulting in a set of 15 vectors (pGB-01;02, − 02;03, − 03;04, − 04;05, − 05;06, − 06;07, − 07;08, − 08;09, − 09;10, − 10;11, − 11;12, − 12;13, − 13;14, − 14;15, − 15;16), each containing unique, but sequentially compatible 5′ and 3′ overhangs after cleavage with BsaI, as shown in Fig. 1.

It was crucial to choose overhangs that differ in at least two positions from all other overhangs in the system. In our initial experiments, we had chosen an overhang for which the reverse-complement sequence differed only in 1 position from another quadruplet, and the efficiency of full assembly dropped to only ~ 10% (data not shown). Similar observations have been made with other Golden Gate systems [16].

Another vector compatible with the Tn7 transposition system with a different antibiotic marker was needed as a destination vector to accommodate the assembled cassettes. For this purpose, we modified the pKL vector, which contains the kanamycin resistance cassette, to include a *ccdB* spacer flanked by BsaI sites in the reverse orientation as compared to the pGB vectors described above, creating overhangs 01 and 16 upon cleavage and at the same time removing the BsaI sites. Additionally, we introduced a PmeI restriction site downstream of the overhang 16 to enable release of the last expression cassette during the analytical restriction screening. This vector has been named pGB-Dest.

To achieve the best possible versatility of the system, we also created two series of “dummy” constructs that can be used as spacers when a smaller number of genes is to be assembled. The first set of 15 dummies contain a short sequence flanked by BsaI sites that create the same overhangs as each individual pGB vector, and therefore can be used to leave out a single position if necessary. In the second set of dummies, each of the available overhangs starting from 03 is paired to 16. In this configuration, it is possible to encompass all remaining empty positions after the last one needed for an expression cassette, in order to create constructs containing less than 15 expression cassettes (Fig. 1).

During the first phase of testing, several components of the proteosomal lid complex from the yeast *Chaetomium globosum* and several human chaperone proteins were cloned together in various combinations. We had previously attempted to express these 10 proteins together from a single baculovirus using the MultiBac™ system, but we failed to succeed in cloning all 10 genes onto a single baculovirus (data not shown). With the pGB vector series, we could create various combinations of these genes by first cloning each gene individually into a specific pGB vector, and then combining all desired expression plasmids into a single construct via a single step Golden Gate reaction. In these initial experiments, the reaction was rather inefficient (1 out of 6 colonies selected contained a correctly assembled clone), but the efficiency was increased to > 90% in later experiments (see Methods and next section).

**Fig. 2:**
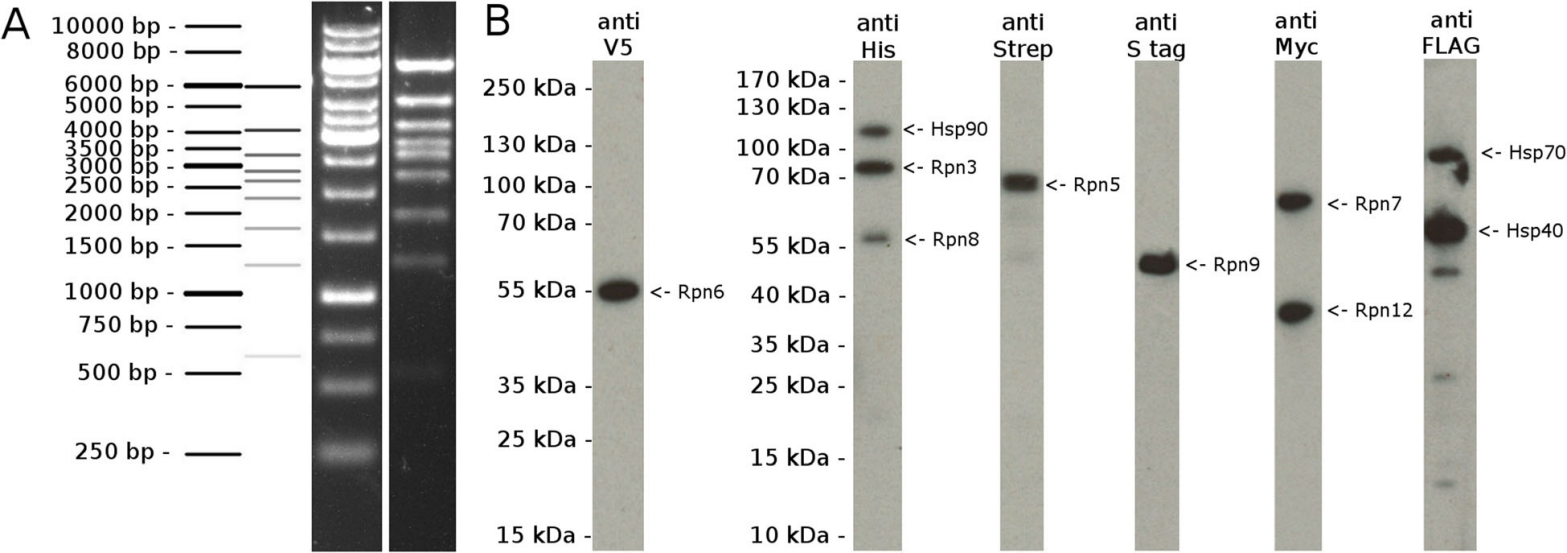
Cloning and production of 10 proteins from a single baculovirus. ***a****.* XhoI digestion of a plasmid containing expression cassettes for the 7 proteins of the proteosomal lid complex plus Hsp40, 70, and 90. The pattern of bands expected for the DNA sizing ladder and the XhoI digest are shown on the left. The actual ladder and digest samples were separated and analyzed via agarose gel electrophoresis and are shown on the right. ***b****.* A baculovirus generated from the clone shown in *A* was used to test whether the proteins from all 10 encoded genes are recombinantly expressed. Western blot analysis of total insect cell lysate using antibodies against epitope tags present on each protein indicates that all 10 proteins are expressed from the single baculovirus

One of the cloned co-expression constructs was chosen to confirm expression of all 10 proteins in insect cells. The presence of all 10 genes in a single construct was verified by XhoI digestion (Fig. 2a). This construct contains the following 10 proteins that have been modified to contain short epitope tags to facilitate identification via Western blot: Rpn3 (66 kDa, N-terminal HIS6), Hsp70 (human HSPA8; 72 kDa, N-terminal FLAG), Hsp40 (human DNAJA1; 46 kDa, N-terminal FLAG), Hsp90 (human HSPAA1; 85 kDa, N-terminal HIS6), Rpn5 (61 kDa, N-terminal TwinStrep), Rpn6 (49 kDa, C-terminal V5 epitope), Rpn7 (56 kDa, N-terminal c-Myc), Rpn9 (46 kDa, C-terminal S•tag), Rpn12 (33 kDa, N-terminal c-Myc) and Rpn8 (40 kDa, N-terminal HIS6). The DNA plasmid analyzed in Fig. 2a was used to generate a single baculovirus, which was then used to infect insect cells to induce recombinant production of the 10 encoded proteins. All 10 proteins were successfully detected in insect cell lysates by Western blot analysis (Fig. 2b).

Once it was clear that the GoldenBac system was efficient and simple to use, we modified the pGB-Dest and all individual pGB entry vectors to be additionally compatible with KO1629-based homologous recombination, thereby creating a new series of vectors called pGBU for “universal”, as these vectors can be used either for Tn7 transposition or homologous recombination. pGBU entry vectors were created by amplification of the BsaI-flanked cassettes from the 15 pGB entry vectors and insertion into a modified pOmniBac1 vector [17], which includes both Tn7 transposase sites for Bac-to-Bac baculovirus generation *and* the ORF603/ORF1629 sites for baculovirus generation via the KO1629-based homologous recombination system. The pGBU-Dest vector was similarly created by the insertion of the ORF603 and ORF1629 ‘flanking’ sites into the pGB-Dest vector (see Methods). Finally, in order to enable virus generation and amplification to be easily monitored by fluorescence, as is possible with the Bac-to-Bac system [5], we constructed a pGBUeGFP-Dest plasmid that contains an eGFP expression cassette downstream of the final Golden Gate cloning site (see Methods). When this plasmid is used to generate a recombinant baculovirus it will constitutively express an un-tagged eGFP reporter enabling simple verification of successful virus generation and amplification, a feature that until now has not been available for users of homologous recombination-based systems. When the same 10 pGB entry modules were inserted into the pGBU-Dest destination vector via Golden Gate, the efficiencies achieved ranged from 60 to 80% (data not shown).

### GoldenBac can be used to efficiently generate large complexes, such as the APC/C

**Fig. 3:**
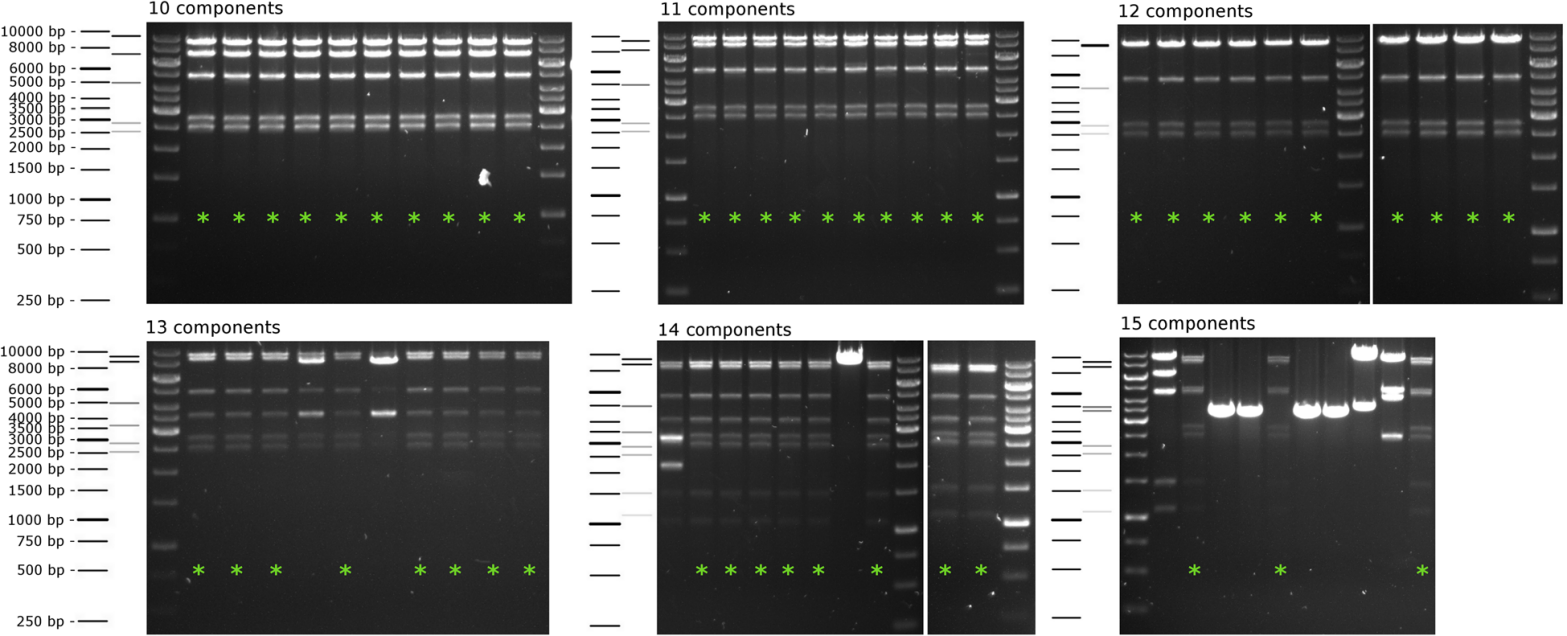
Efficiency of Golden Gate assembly of different APC/C constructs containing 10–15 expression cassettes. To test the efficiency of the GoldenBac system, Golden Gate assemblies were performed using different numbers of expression cassettes. Ten clones resulting from each assembly reaction were picked, digested with EcoRV, and analyzed via agarose gel electrophoresis. A schematic of the DNA sizing ladder and the predicted band pattern for each assembly reaction is shown to the left of the respective agarose gel. Clones demonstrating correct assembly based on the pattern of bands are marked with a green asterisk. The reaction maintains 80% efficiency with assembly of up to 14 expression cassettes. One of the clones containing 14 genes was then used for baculovirus generation and recombinant protein production of the APC/C complex

Having established the system for co-expression of up to 10 proteins, we aimed to test the boundaries of GoldenBac with respect to the efficiency of assembly for up to 15 components. For this, we chose the human anaphase promoting complex/cyclosome (APC/C) as a test system. The APC/C co-expression construct features 14 open reading frames [2]. We cloned each of the components into one of our 14 pGB entry vectors, leaving one empty position to be filled with a dummy during the assembly. With the full APC/C construct, we initially encountered rather low efficiency of assembly (only 1 out of 9 colonies tested were positive after assembly). To more thoroughly test efficiency, the protocol was optimized so that equimolar amounts of the entry plasmids were used (see Methods), and a series of APC/C constructs containing 10–15 components were generated by employing the dummy vectors described above. We tested 10 colonies from each assembly and found the limit of very high efficiency (> 90%) to be at 12 inserts. The efficiency was reduced to 80% with 13–14 components and dropped to 30% with the maximum number of components tested (Fig. 3).

To ensure that these large assemblies also yield functional protein, we used one of the clones encoding all 14 genes making up the APC/C, and produced the entire APC/C complex following a protocol developed using the biGBac system [2]. With biGBac, the highest protein yields after purification of the complex were observed when insect cells were co-infected with two viruses, one containing all APC/C components, and one containing 6 of the components that are part of a subcomplex called the Platform. The Platform construct was easily generated via a single Golden Gate reaction with the 6 platform components, the dummy plasmid 07;16, and the destination vector. In order to be able to distinguish the APC/C and Platform viruses, transposition of the Platform construct was performed using a modified version of the EmBacY bacmid where the YFP marker has been substituted with CFP (BlueBac; see Methods). An additional bacmid that expresses mCherry instead of YFP, called RedBac, was also created. Together with the YFP marker carried by the already widely used EmBacY, the efficiency of infection of up to three viruses can now be monitored simultaneously.

**Fig. 4:**
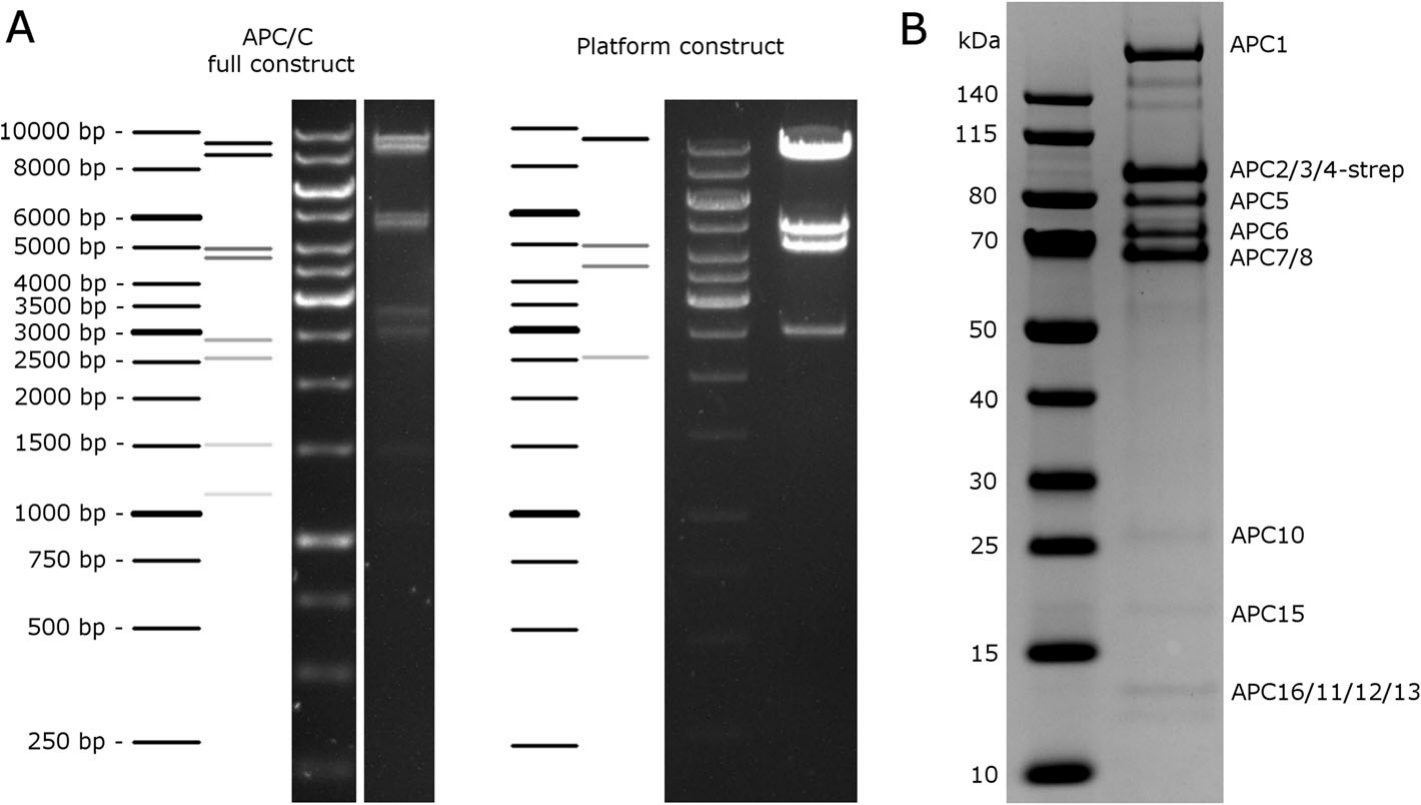
Purification of the APC/C using a GoldenBac generated virus. The APC/C was purified using a three-step procedure consisting of Strep-Tactin affinity chromatography followed by ion exchange and size exclusion. The final protein complex isolated by size exclusion was analyzed by SDS-PAGE on a 4–12% Bis-Tris NuPAGE minigel and stained with Coomassie blue. All components of the APC/C can be identified, as indicated to the right of the gel. Lane 1, molecular weight marker; lane 2, 2 μg of the final purified APC/C complex.

The two constructs, APC/C and Platform, were then subsequently used to create recombinant baculoviruses and the complex was expressed in *Tni* cells, purified and analyzed via SDS-PAGE (Fig. 4). The yield from 0.5 L of insect cell culture containing 2.2 × 10^6^ cells/mL was approximately 70 μg of purified APC/C complex. Average yields of the same purification performed with APC/C and Platform viruses generated using the biGBac cloning system resulted in on average 200 μg of purified complex from 1.2 L insect cell culture containing 2 × 10^6^ cells/mL, demonstrating that viruses generated with the two different cloning systems yield similar amounts of protein ([2]; Fig. 4).

**Fig. 5:**
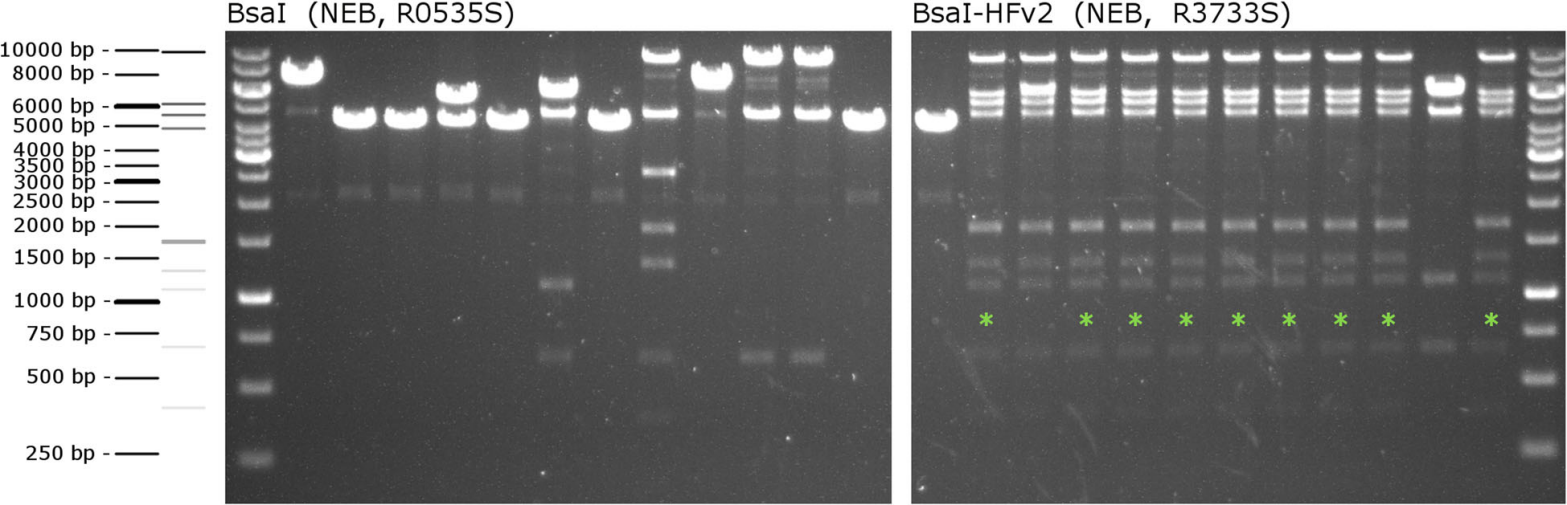
Efficiency of different BsaI enzymes. To test the efficiency of the BsaI-HFv2 enzyme, the same 15 entry vectors were used in a Golden Gate reaction with either the standard BsaI enzyme or BsaI-HFv2. Ten clones resulting from each assembly reaction were picked, digested with EcoRV, and analyzed via agarose gel electrophoresis. A schematic of the DNA sizing ladder and the predicted band pattern for the assembly reaction is shown to the left of the respective agarose gel. Clones demonstrating correct assembly based on the pattern of bands are marked with a green asterisk. While the reaction performed with the standard BsaI enzyme resulted in no correct clones, the reaction with the BsaI-HFv2 enzyme showed 9/10 correct clones

During another especially challenging project where 15 expression cassettes were to be assembled, we compared the influence on efficiency of two BsaI enzyme variants (BsaI from New England Biolabs Cat. No. R0535S and BsaI-HFv2 also from NEB Cat. No. R3733S). The BsaI-HFv2 has greatly enhanced the efficiency of more challenging assemblies. In this particular case, efficiency jumped from 0 positives out of 12 tested when the original BsaI version was used to 9 positive clones out of 12 tested for BsaI-HFv2 (Fig. 5).

### GoldenBac allows for generation of multiple constructs in parallel for screening of co-expression conditions

To further test the applicability of GoldenBac, we used the method to screen different co-expression constructs coding for multiple chaperones for their ability to increase the solubility of hard to express proteins. We chose the human HOIP (HOIL interacting protein), also known as RNF31, an E3 ubiquitin ligase that is a component of the LUBAC complex, as our target protein. We had previously determined that HOIP could be expressed in insect cells, but it remained mainly insoluble (data not shown). We hypothesized that co-expression with chaperones may help increase yields of the HOIP protein. Although baculoviruses up-regulate the expression of specific chaperones upon infection, the up-regulation declines after 6 to 12 h [18, 19]. Moreover, the insect cell Hsp70 proteins play an important role in the viral replicative cycle and might therefore not be available to assist folding of recombinantly expressed proteins [20]. Candidate approaches testing single target proteins and single combinations of chaperones have previously been published, however, most studies use approaches involving co-infection with separate viruses [21–23]. To overcome the drawbacks of co-infection studies and demonstrate the utility of the GoldenBac system for generating constructs for co-expression screening, we designed 10 constructs containing TwinStrep-tagged HOIP (TS-HOIP) together with different combinations of chaperones in a single expression vector (Table 1).

**Table 1:** HOIP-Chaperone combinations created with the GoldenBac cloning system

Construct name	Gene 1	Gene 2	Gene 3	Gene 4	Gene 5
**TS-HOIP**	TS-HOIP	–	–	–	–
**HC1**	TS-HOIP	F-HSPA8	–	–	
**HC2**	TS-HOIP	F-HSPA8	F-DNAJA1	–	–
**HC3**	TS-HOIP	F-DNAJA1	–	–	–
**HC4**	TS-HOIP	F-HSPA8	F-DNAJA1	His-HSPH2	–
**HC5**	TS-HOIP	F-HSPA1A	F-DNAJA1	–	–
**HC6**	TS-HOIP	F-HSPA8	F-DNAJB1	–	–
**HC7**	TS-HOIP	F-DNAJA1	His-DNAJB1	–	–
**HC8**	TS-HOIP	F-HSPA8	F-DNAJA1	His-DNAJB1	–
**HC9**	TS-HOIP	F-HSPA8	F-DNAJA1	His-HIP	His-Hsp90AA1
**HC10**	TS-HOIP	F-sfHSPA8	F-sfDNAJA1	–	–

*TS* TwinStrep affinity tag, *F* FLAG epitope tag, *His* His6 affinity tag, *sf Spodoptera frugiperda*. All genes were expressed under the control of the *polH* promoter, except for DNAJA1, which was under the control of the *p10* promoter

The Hsp70/Hsp40 chaperone system is evolutionary highly conserved between all three domains of life [24, 25]. Therefore, we chose two human Hsp70 chaperones, namely HSPA8 and HSPA1A, and one Hsp70 chaperone endogenous to Sf9 cells, sfHSPA8. The human Hsp70 proteins were combined with human Hsp40 proteins, either DNAJA1 or DNAJB1, whereas the Sf9 chaperone sfHSPA8 was combined with the Sf9 Hsp40 protein sfDNAJA1. Moreover, we constructed co-expression vectors that code for additional human Hsp70-regulating factors, for example HIP (Hsp70 interacting protein), Hsp90AA1 or HSPH2, a member of the Hsp110 family (Table 1). To limit competition for expression factors between the recombinant proteins, as seen in previous studies with Hsp40 [26], human DNAJA1 was cloned into a pGB-3-4 vector in which the *polyhedrin* (*polH)* promoter was exchanged with the *p10* promoter.

**Fig. 6:**
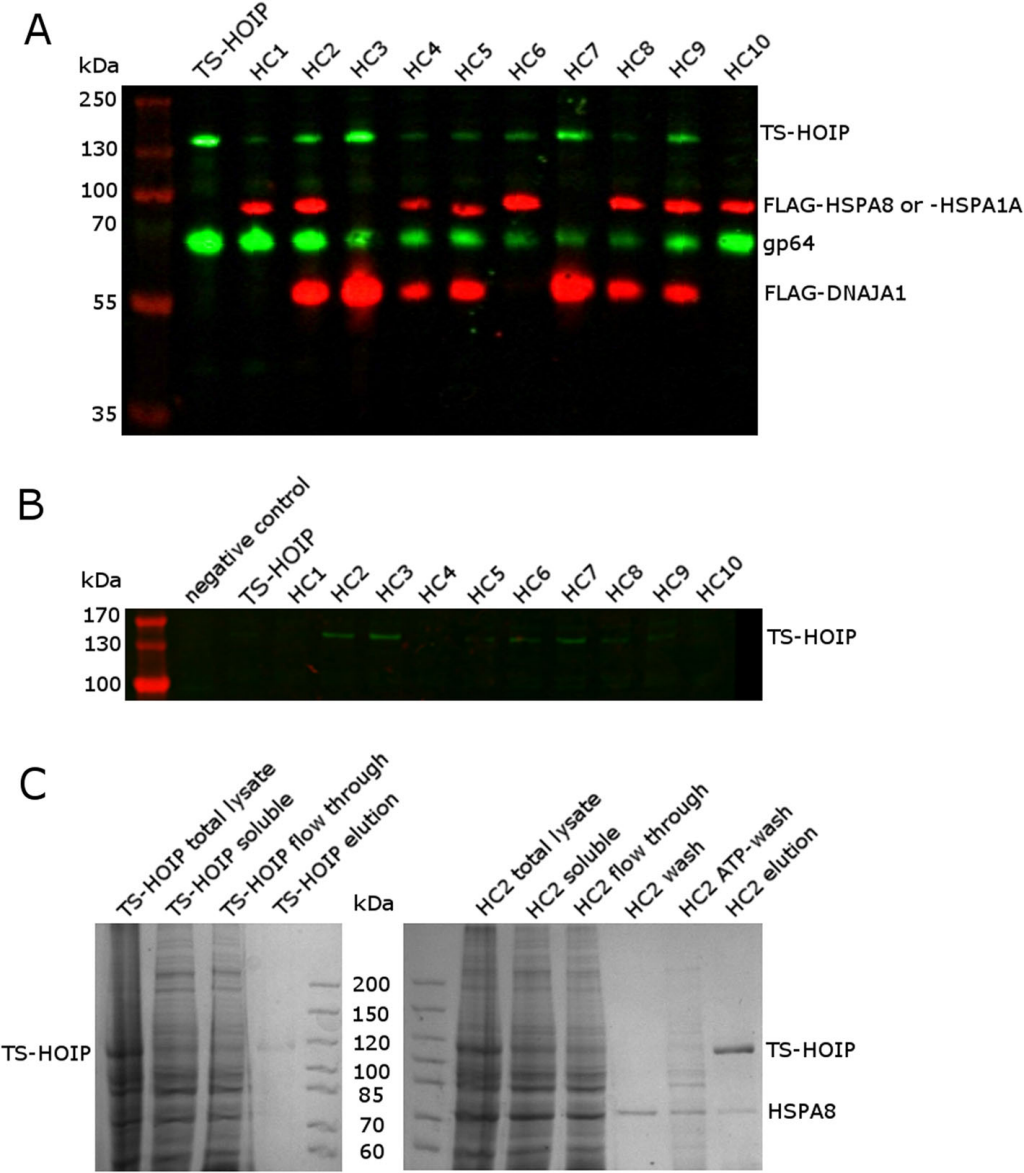
GoldenBac facilitates screening of chaperone co-expression constructs. Ten different baculoviruses expressing the TwinStrep-tagged HOIP (TS-HOIP) protein together with different combinations of chaperones were generated and HOIP expression levels were analyzed. Composition of the constructs HC1–10 are described in Table 1. ***a****.* Total cell lysates were analyzed by Western blotting to detect TS-HOIP and FLAG-tagged HSPA8, HSPA1A, sfHSPA8, or DNAJA1. The baculovirus protein gp64 was used as a loading control. ***b****.* Soluble fractions were also analyzed by Western blotting for TS-HOIP. ***c****.* SDS-PAGE analysis of the purification of TS-HOIP alone (left) or of construct HC2, which contains TS-HOIP co-expressed with HSPA8 and DNAJA1 (right). HSPA8 is visible as a contaminant in the elution

The successful co-expression of HOIP and the various chaperones was verified by Western blotting of total cell lysates. Notably, many of the co-expression trials with Hsp70 proteins, in particular with sfHSPA8, resulted in lower expression levels of HOIP (Fig. 6a). The effect of chaperone co-expression on the amount of HOIP found in the soluble fraction was also qualitatively assessed via Western blot (Fig. 6b). As seen in Fig. 6b, expression of HOIP in constructs HC2, HC3, and HC7 leads to an increase in soluble HOIP as compared to expression of HOIP without chaperones. We chose construct HC2 to further confirm the positive effect of chaperone co-expression by purifying HOIP that was either expressed alone or with the chaperones in HC2 (HSPA8 and DNAJA1). Indeed, a higher amount of soluble protein could be isolated when HOIP was expressed together with HSPA8 and DNAJA1, although despite extensive washing, some HSPA8 remained bound to HOIP (Fig. 6c). This approach illustrates that the modular nature of the GoldenBac system makes establishment of routine screening of co-expression partners, such as chaperones, effector proteins, or subunits of a complex, easily possible for any laboratory specialized on protein production.

## Discussion

Here we demonstrate the utility of the GoldenBac system in generating multi-gene expression constructs with high efficiency. Once the individual expression constructs are ready and cloned into the appropriate pGB vector, multi-gene constructs can be assembled and verified in 3–5 days. We routinely screen only 2 clones per construct to identify a construct containing one copy of each gene, and often both clones are correct. Clones can be screened either by restriction digest, or by Sanger sequencing using one gene-specific sequencing primer per gene to ensure the presence of each cassette. A comparison of GoldenBac and other existing multi-gene expression systems for the baculovirus system is shown in Table 2, and features of the different systems are discussed below.

**Table 2:** Comparison of multi-gene expression systems available for the BEVS

	MultiBac/OmniBac	LIC-based MacroBac	USER-based system	biGBac	GoldenBac
**Number of genes**	up to **4** with Cre-lox, more possible via restriction/ligation	up to **8**, more possible with additional cloning rounds	up to **16** more possible with additional cloning rounds	up to **25**	up to **15**
**Expression of intermediates**	**no**, only those in or fused to acceptors	**yes**	**yes**	**yes**	**yes**
**Efficiency**	Varies; triple assembly via Cre-lox has very low efficiency	**2 colonies** to be screened at each step	**10 colonies** to be tested for assembly of dual cassettes into multi-gene constructs	**6–12 colonies** to be tested in first assembly step	**2 colonies** tested for assembly of up to 12 genes
**Construct verification**	**many possible variants** with possibility of Tn7 element duplication	only **1 correct version** at each step	only **1 correct version** at each step	only **1 correct version** at each step	only **1 correct version**
**Sequence requirement**	2–4, **none** 4^+^, no **BstXI** sites	no **PmeI, SwaI** sites	**none**	2–5, **none** 5^+^, no **PmeI** sites	no **BsaI** sites
**Number of assembly steps**	2–4 genes, 1 step 4^+^, 2 steps	2 genes, 1 step, 4 genes, 2 steps, 8 genes, 3 steps	2–4 genes, 1 step 4^+^, 2 steps and 2 bacmid integrations	2–5 genes, 1 step 5^+^, 2 steps	1 step
**Method of Baculovirus generation**	Tn7 transposase or **homologous recombination**	Tn7 transposase only	Tn7 transposase only	Tn7 transposase only	Tn7 transposase or **homologous recombination**

Information on cloning systems is taken from: MultiBac/OmniBac [17, 27], USER [6], biGBac [2], MacroBac [7]

In comparison to existing systems, GoldenBac shows higher efficiencies and shorter timelines, and therefore also lower costs. While the MultiBac™ system proved revolutionary at the time of its development, it has shown intrinsic limits both in the number of genes that can be combined, and in technical challenges in its application. Due to the reliance on a system of donor and acceptor vectors, many single expression vectors generated via MultiBac™ cannot be used for individual expression. Most important, due to its reliance on assembly via Cre-lox recombination, it is critical to verify the copy number of each gene incorporated into a MultiBac™ construct to ensure the presence of only one copy of the donor vector, and therefore only one set of Tn7 recombination sites, adding a tedious additional verification step that must be customized for each construct generated, which complicates easy and efficient application of the system.

Zhang and colleagues have recently developed a system for multi-gene assembly based on USER cloning [6]. While this system overcomes the need for donor and acceptor vectors, at least two rounds of PCR-based amplification and subsequent assembly are required to combine more than 4 genes. In addition, as the system relies on incorporation of two transfer plasmids into the bacmid, one at the Tn7 site and one at the loxP site, two subsequent transposition steps must be performed.

Another system, called MacroBac, was recently developed and is based on “BioBricks” assembly, using either restriction/ligation reactions or ligation independent cloning (LIC). As with the USER-based system, multiple rounds of assembly are necessary, with three rounds of subcloning required to assemble 8 expression cassettes [7].

The biGBac system, developed by Weissmann and colleagues and based on Gibson assembly, has many advantages: all intermediate constructs can be used for expression, it is modular and amenable to parallel generation of multiple constructs, and it can be used to assemble a very large number of genes (up to 25). Generation of biGBac constructs containing more than 5 genes requires two steps, versus one step required for GoldenBac. In addition, the first of these steps is PCR-based, increasing the expense and decreasing the efficiency of the first Gibson assembly reaction to 33% [2]. However, as the high efficiency of the GoldenBac system starts to decrease when more than 14 genes are combined, for very large expression constructs, biGBac is the system of choice. For constructs containing less than 14 genes, the high efficiency of GoldenBac is a great advantage.

GoldenBac and other multi-gene expression systems rely almost exclusively on the *polH* promoter, as this is the strongest baculoviral promoter currently available. Although all genes in the proteasomal lid complex and APC/C constructs used in this study are controlled by the *polH* promoter, individual gene expression levels vary, as evidenced by the necessity to express some of the components of the APC/C in two copies. This is a phenomenon that has been observed in other multi-gene expression systems as well [2, 3]. In these cases, differences in expression levels are most likely due to intrinsic differences in the gene sequences, and it is therefore difficult to draw any conclusions regarding the effect of multiple identical promoters on gene expression.

The presence of multiple copies of the *polH* promoter sequence in multi-gene constructs may also influence viral stability. Over the course of this study, we did not observe any detrimental effects resulting from multiple use of the *polH* promoter, however, we were not able to analyze viral stability over a time period of more than a few months. To avoid problems with viral stability, it is advisable to adhere to the practices outlined previously for multi-gene expression constructs generated by the MultiBac™ system [27], including starting with healthy, low passage insect cell cultures exhibiting high viability, and keeping viral amplification times as short as possible to avoid accumulation of defective viral particles. However, we did note that for multi-gene constructs with very large inserts, such as the full APC/C construct, which has a total insert size of 33.2 kbp and was the largest construct generated in this study, typical virus amplification times had to be extended by approximately 24 h, perhaps due to slower packaging of such large baculoviral genomes. In the future, it may be possible to optimize expression and stability of such large constructs through the use of synthetic baculovirus genomes [28], which will be smaller and more stable, and should therefore effectively counteract issues surrounding the use of extremely large inserts.

## Conclusions

We describe the development of a robust, single-step cloning system for generation of multi-gene expression constructs. The system allows for efficient and cost-effective assembly of expression plasmids containing up to 15 open reading frames. Verification of correct incorporation of all expression cassettes can be quickly performed by restriction digest. The system has been adapted both for Tn7 transposition and recombination-based baculovirus generation technologies. The high efficiency and low cost of the system makes it ideal for introduction into the standard workflows of core facilities or any laboratory that requires routine production of protein complexes.

## Methods

### Generation of GoldenBac vectors

In order to create the pGB vector series for single protein expression that also allow for later combination into co-expression constructs, pACE-Bac1 [29] has been modified to contain a *ccdB* spacer for negative background selection that has been amplified from pCoofy31 [30], including the 3C-cleavable N-terminal TwinStrep tag and the C-terminal His6 tag. Two BsaI sites were introduced to flank the expression cassette and the 4 bp that form an overhang upon cleavage were designed to form one of 16 unique quadruplets 01–16, so that the overhang downstream of the SV40 terminator in one vector will be compatible with the overhang upstream of the *polH* promoter in the next vector. Additionally, the cryptic start (ATT) in the *polH* promoter upstream of the start codon that is known to be partially active (described in the ThermoFisher Scientific Bac-to-Bac manual and occasionally observed in our early work, data not shown) has been rendered out of frame of the GOI by inserting an additional adenine towards the end of the promoter in the pGB vector series. To generate the pGB-Dest vector, the pKL vector [27] was modified to include a *ccdB* spacer flanked by BsaI sites in the reverse orientation as compared to the pGB vectors described above that has been amplified from the pGGZ003 vector [16], and a PmeI site was introduced downstream of the overhang 16.

pGBU entry vectors were created by first removing one of the two AgeI sites and the BsaI site (positions 713 and 1846, respectively) from the pOmniBac1 [8] (Geneva Biotech) vector backbone using Quikchange mutagenesis (Agilent). The resulting ΔAgeI-ΔBsaI pOmniBac1 vector was cut with DraIII and AgeI enzymes. The BsaI-flanked expression cassettes from the 15 pGB entry vectors were amplified by PCR and inserted into the cut ΔAgeI-ΔBsaI pOmniBac1 plasmid using In-Fusion cloning (Takara Biosciences). The resulting pGBUniversal entry vectors were sequence verified and tested for *ccdB* functionality before use.

The pGBU-Dest plasmid was created by the sequential insertion of the ORF603 and ORF1629 flanking sites into the pGB-Dest plasmid. First, a SapI-EcoRV fragment containing the *lef2* and ORF603 genes and the Tn7R transposase site was amplified from the ΔAgeI-ΔBsaI pOmniBac1 plasmid. The resulting fragment was then cloned into pGB-Dest linearized with SapI and EcoRV enzymes by In-Fusion cloning. Correct insertion of the fragment was confirmed by sequencing before the insertion of the Tn7L/ORF1629 fragment. Second, a fragment containing the Tn7L transposase site and ORF1629 was amplified from pOmniBac1 and the resulting PCR product was first purified and reamplified to add an extension from the pGB-Dest backbone to enable In-Fusion cloning into the previously prepared pGB-Dest+ORF603 plasmid cut with PmeI and AsiSI.

The pGBUeGFP-dest plasmid was created by insertion of an expression cassette containing an untagged eGFP gene (from an existing pGB entry vector construct) downstream of the final BsaI cloning site of the pGBU-Dest plasmid, using In-Fusion cloning into pGB-Dest cut with PmeI. Both pGBU-Dest and pGBUeGFP-dest plasmids were sequence verified and tested for *ccdB* functionality before use.

### Generation of pGB or pGBU individual expression constructs

The GoldenBac vectors are compatible with the RecA-mediated Sequence and Ligation Independent Cloning strategy [30], Gibson Assembly [31], or In-Fusion cloning (Takara Biosciences). Target genes were amplified from existing plasmid DNA templates or cDNA using Phusion Flash HiFi polymerase (ThermoFisher Scientific) and primers containing 20–24 nt overhangs allowing for insertion into any pGB/pGBU vector, with the desired epitope or affinity tag added onto the primer if required. pGB/pGBU vectors were amplified via PCR using the corresponding compatible primers. PCR products and linearized, amplified vectors were gel purified using the GeneJET Gel Extraction kit (ThermoFisher Scientific). 0.025 pmol of the desired vector and 0.1 pmol of the desired insert were combined with 1 μl RecA protein and 1 μl RecA reaction buffer (New England Biolabs), in a final volume of 10 μl. The reactions were incubated at 37 °C for 30 min. The entire reaction was transformed into chemically competent NEB10beta *E. coli* cells (New England Biolabs) and clones were selected on gentamicin-containing LB agar plates. The DNA sequence of the inserted genes were verified by Sanger sequencing.

For In-Fusion cloning of inserts into pGB or pGBU entry vectors, target genes were amplified with 15 bp extensions compatible with the appropriate termini of the linearized plasmids. Template DNA was removed by treatment with DpnI restriction enzyme (ThermoFisher Scientific) and, unless multiple PCR product sizes were observed, a simple reaction clean-up step (Ampure from Beckman Coulter) with elution in water or 10 mM Tris-HCl (pH 8.5) was used in place of gel purification. Ten microliter standard In-Fusion (Takara Biosciences) reactions containing 100 ng of linearized plasmid and 100 ng of insert were incubated for 30 min at 42 °C, immediately diluted 1:5 with TE buffer (10 mM Tris-HCl, pH 8.0, 1 mM EDTA) and 5 μl were used to transform chemically competent Omnimax2 cells (ThermoFisher Scientific), with clone selection on gentamicin-containing LB agar plates.

### Multi-gene assembly via the Golden Gate reaction

For the Golden Gate reaction, initially 250 ng of each of the desired modules were mixed with 100 ng of the destination vector, 2 μl T4 DNA ligase buffer (ThermoFisher Scientific), 1.2 μl T4 DNA ligase (ThermoFisher Scientific) and 1 μl BsaI (New England Biolabs) and 0.2 μl BSA (New England Biolabs) in a total volume of 20 μl. In later experiments, the efficiency of the reaction was increased by combining equimolar amounts of modules (0.05 pmol) with 0.025 pmol of destination vector. The following program was then run on a thermocycler T100 or C1000 (BioRad): 50 cycles of 37 °C for 5 min and 16 °C for 5 min, followed by 37 °C for 30 min, 50 °C for 5 min, and 80 °C for 5 min. After the reaction was finished, 1 μl PlasmidSafe nuclease (Epicentre) and 0.85 μl of 25 mM ATP were added to each reaction, and the reactions were incubated at 37 °C for 60 min followed by 70 °C for 30 min, to remove any intermediate, not fully ligated products. 3 μl of each reaction were then transformed into chemically competent NEB10beta cells (New England Biolabs) and correct clones were selected on kanamycin-containing LB agar plates. Clones were analyzed via PmeI digestions and/or Sanger sequencing to confirm the presence of each individual expression cassette.

### Creation of the modified bacmids “BlueBac” and “RedBac”

In order to obtain new baculoviral backbones bearing fluorescent markers that could be detected alongside the YFP of the EmBacY, the mCherry and CFP genes were first separately cloned into the pIDC vector [29]. Bacmid DNA containing no fluorescent marker was purified from DH10MultiBac cells [4] by alkaline lysis followed by isopropanol precipitation. The bacmid DNA was then fused to the CFP_pIDC or mCherry_pIDC construct via in-vitro Cre-lox recombination using the Cre recombinase (M0298S, New England Biolabs). The reaction was then osmotically desalted in a mold made of 1% agarose + 100 mM glucose and the DNA was electroporated into NEB10beta cells (New England Biolabs) using the low voltage protocol on the BioRad GenePulser device. Cells were then selected on LB agar plates containing kanamycin and chloramphenicol. Since the pIDC vector carries a conditional origin of replication, oriR6Kγ, which requires a *pir* + strain for propagation, only cells that carry the pIDC construct fused to the bacmid can survive. For each construct, 1 colony was then expanded in LB containing kanamycin and chloramphenicol and made electrocompetent. The helper plasmid that encodes the transposase and thus provides the Tn7 transposition function *in trans* was purified using from the DH10MultiBac cells, electroporated into the cells carrying the new bacmids, and selected on LB agar plates with kanamycin, chloramphenicol and tetracycline. A resistant colony was then expanded and made chemically competent, thus giving rise to the BlueBac and RedBac cells, respectively. The strains were then tested for functionality both in transposition of transfer plasmid, in generation of baculovirus, and in expression of the fluorescent marker by monitoring fluorescence in the corresponding wavelength.

### Protein expression and purification

Expression vectors were transformed into the *E. coli* strain DH10EmBacY ([5]; Geneva Biotech) or BlueBac. Clones with the successfully integrated expression cassette were selected by blue-white selection on LB agar plates containing X-Gal and IPTG in addition to selective antibiotics. Bacmids were isolated from positive clones and transfected into 1 × 10^6^ Sf9 cells (Expression Systems) per well in a 6-well plate using 3 μg bacmid and 10 μl FuGENE® HD Transfection Reagent (Promega) as described by the manufacturer. The virus backbone encodes a YFP gene, which allows for monitoring of the transfection efficiency by visual observation of fluorescent cells. After 5–7 days, the supernatant containing the first virus generation, V0_,_ was harvested. One millilitre of V0 was used to infect 50 ml Sf9 cell culture at a density of 1 × 10^6^ cells/ml. Fluorescence was measured using a Varioskan Flash Plate Reader (ThermoFisher Scientific). When RFU was above 50, the supernatant, containing the virus generation V1, was harvested. V1 was used for protein expression at a dilution of 1:500.

The proteosomal lid complex and APC/C were expressed in *Tni* cells (Expression Systems) at 27 °C in ESF921 media (Expression Systems). A culture of 50 ml with a density of 1.5 × 10^6^ cells/ml was infected with V1 at a dilution of 1:500. Two days after proliferation arrest, cells from 1 ml of culture were harvested by centrifugation at 150 g for 10 min and stored at − 20 °C.

The APC/C was purified essentially as described [32]. In brief, APC/C, which is expressed with a Twin-Strep-tag on the C-terminus of APC4, was purified by affinity to Strep-Tactin Sepharose (IBA), then by ion exchange, and finally by SEC.

TS-HOIP constructs were expressed in Sf9 cells at 27 °C using ESF921 media (Expression Systems). For Western analysis, a culture of 50 ml with a density of 1.5 × 10^6^ cells/ml was infected with V1 at a dilution of 1:500. Two days after proliferation arrest, cells from 1 ml of culture were harvested by centrifugation at 150 g for 10 min and stored at − 20 °C.

For purification of HOIP, 500 ml of insect cell culture was expressed under the same conditions. The cells were lysed by freeze-thaw in a lysis buffer containing 100 mM Tris pH 8.0, 150 mM NaCl, and 100 μM ZnCl_2_. Cell debris was separated via centrifugation, and the resulting supernatant was loaded onto a 1 ml Strep-Tactin affinity column (IBA). After washing with lysis buffer containing 2.5 mM ATP and 10 mM MgCl_2_, the HOIP protein was eluted in lysis buffer containing 5 mM desthiobiotin (ThermoFisher Scientific). Samples of total cell lysate, supernatant, wash, and elution fractions were analyzed via SDS-PAGE and the gel was stained with InstantBlue gel stain (Sigma-Aldrich).

### Western blot analysis

For the analysis of the HOIP-chaperone co-expression experiments, the frozen cell pellets were resuspended in PBS to a cell density of 1 × 10^6^ cells/ml. A sample of this whole cell lysate (sample T, total) was mixed with 6 x SDS loading buffer (1.67% SDS, 5% glycerol 58 mM Tris-HCl pH 6.8, Coomassie Brilliant Blue). To isolate the soluble fraction of the lysate, 200 μl of the whole cell lysate were centrifuged at 21000 g for 10 min. A sample of the supernatant (sample S, soluble) was mixed with 6 x SDS loading buffer. 5 μl of samples T and 10 μl of samples S of each test expression were loaded onto NuPAGE™ Novex™ 4–12% Bis-Tris Protein gels (ThermoFisher Scientific) and were electrophoresed in NuPAGE® MES SDS Running Buffer (ThermoFisher Scientific). The proteins were transferred onto a PVDF membrane using the Trans-Blot® Turbo™ Transfer System (High MW program) and Trans-Blot® Turbo™ Midi PVDF Transfer Packs (Biorad). Expression of HOIP was detected with an anti-Strep primary antibody (StrepMAB-Classic from IBA) and a fluorescently tagged anti-mouse secondary antibody (IRDye® 800CW Goat anti-Mouse IgG (H + L) from Li-COR). The chaperones were detected with an anti-FLAG primary antibody (Sigma-Aldrich, F7425) and a fluorescently tagged anti-rabbit secondary antibody (IRDye® 680RD Goat anti-Rabbit IgG (H + L) from Li-COR). The gp64 protein was detected with an anti-gp64 primary antibody (LifeSpan BioSciences; LS-C107068) and a fluorescently tagged anti-mouse secondary antibody (IRDye® 800CW Goat anti-Mouse IgG (H + L) from Li-COR). Fluorescence was detected using the Odyssey™ Imaging System (LI-COR).

For the Western blot analysis of the *Chaetomium globosum* proteosomal lid complex, sample preparation, electrophoresis and transfer were performed as described above. Tagged proteins in the whole cell lysate were detected using the following primary antibodies: V5 Tag Monoclonal Antibody (R960–25, ThermoFisher Scientific; mouse), PentaHis Antibody (34,660, Qiagen; mouse), StrepMAB-Classic (2–1507-001, IBA; mouse), Anti-S•Tag (MAC112, Merck; mouse), Myc-Tag 9B11 (2276S, Cell Signaling Technology; mouse), ANTI-FLAG antibody (F7425, Sigma Aldrich; rabbit). Secondary antibodies used were HRP-conjugated Anti-mouse IgG (7076S, Cell Signaling Technology) or Anti-rabbit IgG 1:10000 (7074S, Cell Signaling Technology) and the signal was developed on Amersham Hyperfilm ECL (28,906,835, GE Healthcare) using Pierce ECL Plus Substrate (32,132, ThermoFisher Scientific).

## Data Availability

The full set of pGB vectors has been deposited at the Belgian Coordinated Collection of Microorganisms (BCCM), BCCM/Gene Corner in Ghent, Belgium (http://bccm.belspo.be/catalogues/genecorner-plasmids-catalogue-search). The plasmids can be ordered from the catalog, under accession numbers 11997–12039. A detailed protocol has been posted on the website of the Protein Production and Purification Partnership in Europe (P4EU) website (https://p4eu.org/molecular-biology).

## Abbreviations

BEVS: Baculovirus expression vector system
PCR: Polymerase chain reaction
APC/C: Anaphase promoting complex/cyclosome
Hsp: Heat shock protein
HOIP: HOIL interacting protein
polH: polyhedrin
